# Simulation Study on the Charge Collection Mechanism of FinFET Devices in Single-Event Upset

**DOI:** 10.3390/mi15020201

**Published:** 2024-01-29

**Authors:** Hongwei Zhang, Yang Guo, Shida Wang, Yi Sun, Bo Mei, Min Tang, Jingyi Liu

**Affiliations:** 1 College of Computer Science and Technology, National University of Defense Technology, Changsha 410073, China; onlyweioicq@126.com; 2 China Academy of Space Technology, Beijing 100094, China; wsd980124@163.com (S.W.); zoesun99@126.com (Y.S.); malboo@126.com (B.M.); 13501009044@139.com (M.T.); evoltime@163.com (J.L.)

**Keywords:** FinFET, SRAM, charge collection, bipolar amplification effects

## Abstract

Planar devices and FinFET devices exhibit significant differences in single-event upset (SEU) response and charge collection. However, the charge collection process during SEU in FinFET devices has not been thoroughly investigated. This article addresses this gap by establishing a FinFET SRAM simulation structure and employing simulation software to delve into the charge collection process of FinFET devices during single-event upset. The results reveal substantial differences in charge collection between NMOS and PMOS, and that direct incidence of PMOS leads to the phenomenon of multiple-node charge collection causing SRAM unit upset followed by recovery.

## 1. Introduction

The essence of single-event upset (SEU) is charge collection at the sensitive nodes, triggering a voltage upset when enough charge is accumulated. Prior research has extensively investigated charge collection during SEU in planar devices [1,2,3,4]. However, with the continuous evolution of semiconductor processes, FinFET technology, owing to its superior gate control capabilities, has gradually replaced planar processes in the integrated circuit industry.

The concept of FinFET transistors was first proposed in 1980s as double-gate FETs [5]. FinFET technology was first applied in the commercial sector in 2011 when Intel achieved mass production of the 22 nm FinFET process [6]. This implementation led to significant improvements in performance, striking a better balance between power consumption and heat generation. Consequently, processors utilizing this technology became more suitable for use in laptops and desktop computers. Following Intel’s lead, major foundries established their own FinFET production lines, guiding the continued development of semiconductor devices along the FinFET technology path. Currently, the TSMC (Taiwan Semiconductor Manufacturing Company, Hsinchu, Taiwan) has advanced the improved FinFET technology to the 3 nm process node [7].

The alteration in process structure may lead to changes in charge collection mechanisms. For FinFET devices, the introduction of a narrow fin structure significantly distinguishes the charge collection process from planar devices [8,9,10]. Moreover, comprehensive studies on the single-event effects’ charge collection process in single-fin FinFET devices have been conducted [9,11,12]. However, the charge collection process in FinFET SRAM units differs substantially from single-transistor FinFET devices due to factors such as coupling with neighboring devices and external circuits during single-event upset.

**Figure 1:**
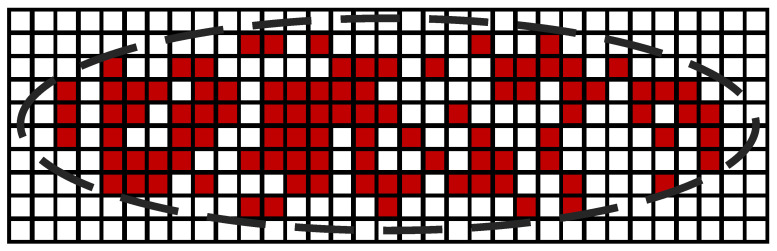
Physical distribution of MCU in SRAM arrays horizontally corresponds to the bitline direction and vertically corresponds to the wordline direction [17].

Static Random-Access Memory (SRAM) is the primary subject of study for single-event upset. SRAM units typically consist of six transistors forming a bistable flip-flop, capable of switching between stable “0” and “1” states. When a heavy ion strikes the drain region, or its vicinity, of a charged NMOS or PMOS in a set state within an SRAM unit, a substantial amount of charge accumulates in this region. The built-in electric field in this region leads to transistor collection of a large amount of charge, causing the transistor to conduct and altering the stored information in the SRAM unit. This process is considered a single-event upset [13]. Sensitive nodes (transistors in a set state) exhibit four charge collection mechanisms during the process: drift, diffusion, parasitic bipolar amplification effects, and well-collapse source injection indirect charge collection [14]. These mechanisms, either independently or collectively, lead to the accumulation of sufficient charge at the sensitive nodes, completing the single-event upset. Single-event upset (SEU) phenomena in SRAM have been observed since the 1970s [15,16]. As technology advances and integration levels increase, the issue of multiple-bit upset (MBU) in FinFET devices has become increasingly severe. In experiments on 5 nm FinFET SRAM devices, the proportion of multiple-bit upsets caused by a single particle even exceeded 90%, with a single particle impact triggering up to 28 upset bits [17]. In SRAM layout design, SRAM units along the bitline direction are located in the same well region, while SRAM units along the wordline direction are in different well regions. Due to the easier diffusion of charges generated in the substrate after particle impact in the same well region, whether for planar devices or FinFET devices, single-event multiple-cell upset (MCU) is always most severe when particles enter along the bitline direction. Figure 1 illustrates the MCU situation caused by a single heavy ion impact, with each box representing an SRAM unit, and red boxes indicating logic bit flips in the SRAM. The elliptical distribution of the flipped SRAM array is evident in the figure due to the well distribution. However, it is observed that not all of the cells within this region were observed to be upset. While charge collection may not upset all cells near the edge of the cluster, there are many cells near the particle strike location that do not show data corruption. Furthermore, experiments conducted on FinFET SRAM, specifically investigating multiple-bit upsets caused by single particles, also suggest the involvement of parasitic bipolar amplification effects [18,19]. Therefore, it is essential to perform simulation modeling for FinFET SRAM devices, analyzing the charge collection process of small-sized SRAM units under single-event effects. This approach contributes to a better understanding of the underlying principles governing charge collection in FinFET SRAM devices.

In this study, TCAD simulation tools were employed to model FinFET SRAM units. The research focused on investigating the single-event upset response process of FinFET SRAM units. Detailed analysis of the charge collection principles during single-event upset in FinFET SRAM units was conducted by extracting the time-dependent variation of the current at the sensitive nodes following heavy-ion impact.

## 2. TCAD Simulation Model

Based on the SPICE model parameters provided by the PTM website [20] and information on FinFET technology described in the literature [21], a three-dimensional FinFET SRAM device model was established using Synopsys’ Sentaurus TCAD (vO-2018.06-SP2) tools through a structural and principled modeling approach [22]. First, use the SDE (Sentaurus Structure Editor) tool to build the structural model of the device, ignoring the actual physical processes generated by the device, and without considering physical quantities such as time and temperature [23]. Next, specify the region for concentration doping, then divide the electrodes and calculate the required grid points. The structural information of the device (such as junction depth, gate oxide thickness, channel length, etc.) can be obtained from the Process Design Kit (PDK) files provided by manufacturers or the publicly available Predictive Technology Model (PTM) from Arizona State University [20]. Subsequently, use the SDEVICE (Sentaurus Device) tool to define the physical models required for the device itself, as well as the initial state and excitation of the device [24]. Finally, based on the structural model, utilize numerical analysis methods to compute the solution of the “physical model”, thereby analyzing the internal physical processes and characteristics of complex devices. The added physical models to this model include: (1) the drift-diffusion model; (2) the Auger recombination model; (3) the SRH indirect recombination model; (4) the bandgap narrowing effect model; (5) the high-field mobility saturation model; (6) the ionized impurity scattering model; (7) the phonon scattering model; (8) the interface scattering model.

**Figure 2:**
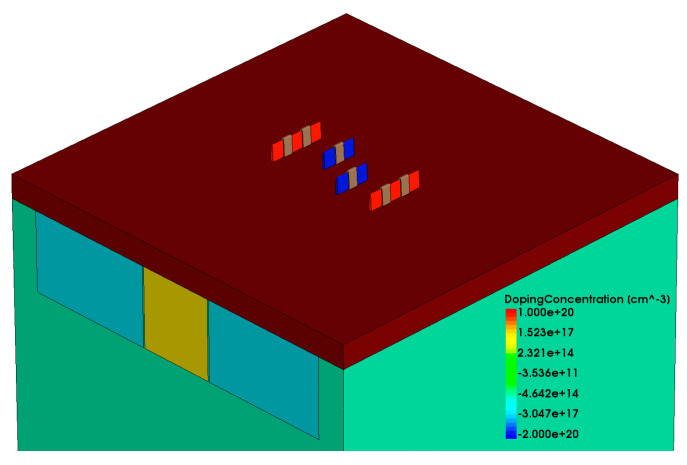
SRAM Simulation Models Based on FinFET Technology.

**Figure 3:**
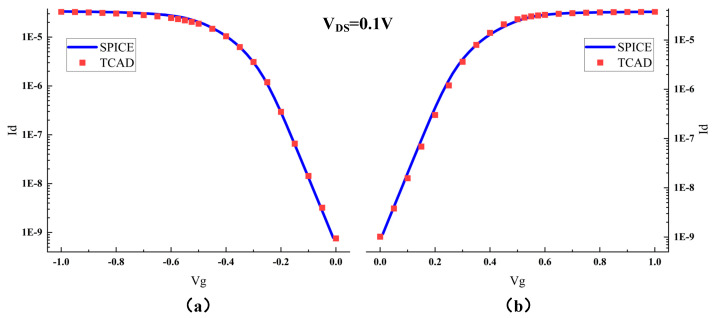
Calibration Curves of TCAD Model and SPICE Model: (**a**) PMOS; (**b**) NMOS.

The established model, as depicted in Figure 2, includes the six transistors required for an SRAM, with the circuit connections between transistors represented by SPICE statements in the metal wiring layer. The main parameters of the device are provided in Table 1. After completing the three-dimensional FinFET device modeling, further calibration of the electrical parameters of the MOS transistors in the model was necessary to ensure accuracy during the simulation process. The SPICE model parameters used for calibration were provided by the PTM website. By appropriately modifying doping concentrations and adding a small amount of trap charge, the calibrated Id–Vg curve of the MOS transistor is shown in Figure 3 [25,26]. The calibrated Id–Vg curve aligns well with the SPICE model curve, indicating favorable fitting and providing the necessary conditions for conducting radiation effects simulation work.

In the physical processes within a semiconductor, the relationship between charge distribution and potential is described using the Poisson equation [27]. The Poisson equation characterizes the spatial variation of charge and potential in space:

$$\nabla^{2}\varphi =-\frac{q}{\epsilon }\left(N+p-n\right)$$

where *φ* represents the electric potential, *N* is the net doping concentration, and *p* and *n* are the hole and electron concentrations, respectively. Additionally, due to the conservation of charge carriers, a given infinitesimal volume in space must also satisfy the charge carrier continuity equation:

$$\frac{\partial n}{\partial t}=\frac{1}{q}\nabla \cdot \vec{J_{n}}+\left(G-R\right)$$

$$\frac{\partial p}{\partial t}=-\frac{1}{q}\nabla \cdot \vec{J_{p}}+\left(G-R\right)$$

where *G* represents generation, *R* represents recombination, and $\vec{J_{n}}$ and $\vec{J_{p}}$ denote the carrier transport equations for electrons and holes (considering only drift and diffusion processes), respectively.

$$\vec{J_{n}}=qD_{n}\nabla n-nq\mu_{n}\nabla \varphi$$

$$\vec{J_{p}}=-qD_{p}\nabla p+pq\mu_{p}\nabla \varphi$$

where $D_{n}$ is the electron diffusion coefficient, ∇n is the gradient of electron concentration, $\mu_{n}$ is the electron mobility, and ∇φ is the potential gradient. By specifying boundary conditions, TCAD will solve the three field equations mentioned above, obtaining solutions for the potential, electron, and hole concentrations within a specific region. Additionally, Sentaurus TCAD incorporates a heavy-ion model, allowing the introduction of a heavy-ion model at specific locations in the device. Parameters such as the ion’s penetration depth, linear energy transfer (LET), and incident direction can be set, enabling the simulation of single-event effects in semiconductor devices.

The top view of the SRAM cell and its circuit connections are shown in Figure 3. Before starting transient simulations, the storage state Q of the SRAM cell is set to 0. At this point, the sensitive nodes for single-event upset are the N1 transistor and the P2 transistor. All simulations in the article are conducted with direct incidence on the sensitive nodes. The incidence radius is set to 10 nm, and the incidence direction is perpendicular. The heavy-ion impact positions are selected at the drain center region of the N1 transistor and the P2 transistor. The corresponding linear energy transfer (LET) thresholds for the NMOS transistor in the SRAM model are approximately 0.8 MeV·cm^2^/mg, and for the PMOS transistor, the LET threshold is approximately 2.3 MeV·cm^2^/mg. In simulations, the unit of Linear Energy Transfer (LET) is expressed as pC/μm, with the commonly used conversion factor of MeV·cm^2^/mg being 1 pC/μm = 96.61 MeV·cm^2^/mg.

## 3. Simulation Results and Analysis

### 3.1. Charge Collection in NMOS Transistor

**Figure 4:**
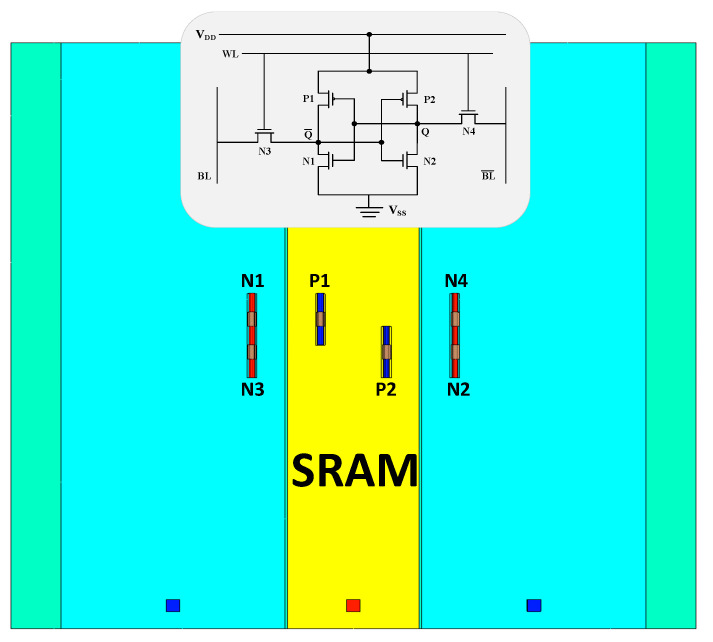
Top View of the SRAM Model (the STI is hidden) and Circuit Interconnect.

With the storage state Q set to 0, the drain region of the N1 transistor becomes the sensitive area. Investigating the response of the N1 transistor at low LET values, the transient drain current near the LET threshold is shown in Figure 4. Before any upset occurs, the transient pulse width and peak of the drain current increase with an increasing LET. However, when the LET reaches 0.009 pC/μm, the transient pulse width of the drain current starts to exhibit a decreasing trend. This is because, for a standard SRAM cell, the minimum LET required to induce single-event upset is related to its critical charge value. With the critical charge usually being a fixed value under constant operating voltage, as the LET increases and the transient peak rises, the collected charge at the drain of the N1 transistor reaches the critical charge value earlier, leading to the closure of the NMOS transistor. This trend manifests as an increase in peak and a decrease in pulse width.

Additionally, around the LET value that can induce SRAM upset, the single-event transient exhibits three peaks. The first peak is due to the direct incidence of particles on the drain-sensitive node, causing some charge to already exist at the drain node without going through any charge collection process. The absorption of these charges weakens the reverse-biased state of the drain-body pn junction, leading to a decrease in the speed of charge collection during the drift process. When analyzing the second and third peaks, we assume that during subsequent processes, charges ionized near the drain-body pn junction will be collected at the drain node through diffusion by the built-in electric field. These charges, along with the remaining charges at the drain node, are slowly absorbed by the drain node, forming the second peak. Furthermore, the presence of the feedback circuit in the SRAM unit itself is the main reason for the third peak. The validity of the assumptions for analyzing the second and third peaks will be detailed below.

**Figure 5:**
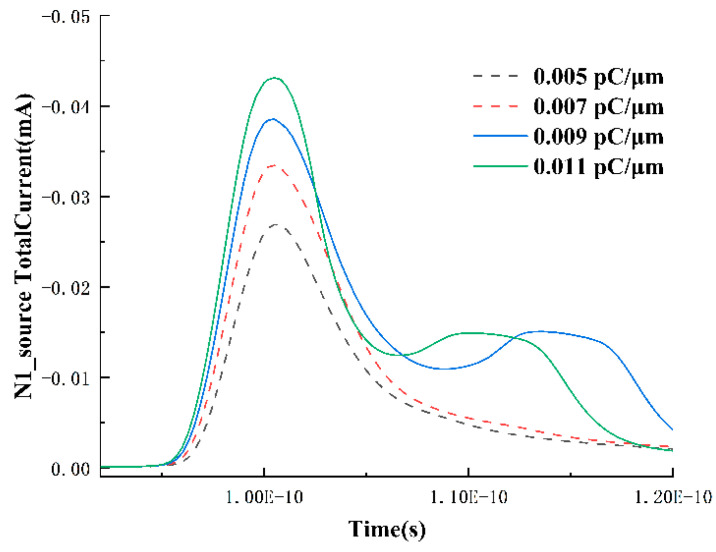
The source current of the N1 transistor and the variation in drain voltage over time at 0.009 pC/μm.

In the occurrence of single-event upset in the SRAM unit, there is a competitive mechanism: excess charge carriers collected at the drain tend to cause a bit flip, while the feedback circuit inherent in the SRAM unit tends to restore the stored data [13]. To verify that the current from the feedback circuit in the SRAM unit also plays a role, the source current of the N1 transistor and the variation in the drain voltage over time at 0.009 pC/μm are extracted, as shown in Figure 5. As the LET value increases, the peak of the source current gradually rises, and at 0.007 pC/μm, it even exhibits a perfect pulse peak. However, when the LET value is raised to 0.009 pC/μm, a distinct secondary peak appears immediately, and the peak value of the secondary peak decreases and occurs earlier with the increase in LET, while the growth trend of the main peak remains unchanged. Under the premise that the charge collected by the source due to diffusion is negligible at lower LET values, it can be inferred that the formation of the secondary peak in the source is due to the feedback mechanism of the SRAM circuit. Even if the voltage at the drain has not dropped to 0 V after heavy-ion incidence, once it enters the low-voltage noise margin region of the SRAM unit, the circuit feedback mechanism will eventually complete the flip from 0.8 V to 0 V.

**Figure 6:**
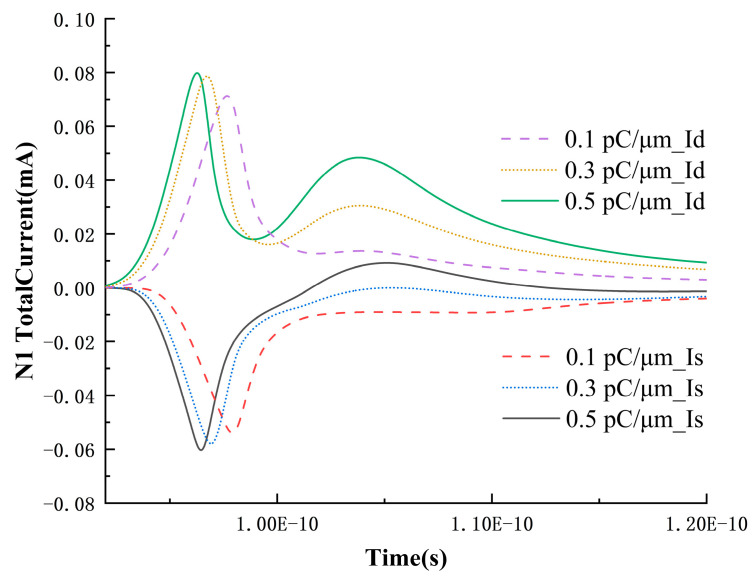
Variation in N1 drain current over time for LET values of 0.1, 0.2, 0.3, and 0.5 pC/μm.

**Figure 7:**
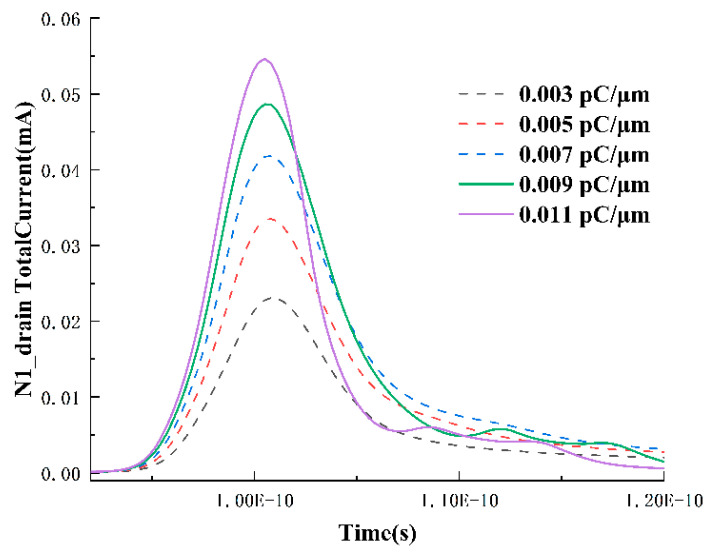
The Variation in Drain Current in N1 Transistor over Time for LET values ranging from 0.003 to 0.0011 pC/μm.

To further determine the physical meanings represented by each peak, the drain and source currents of N1 at 0.1, 0.3, and 0.5 pC/μm are extracted, as shown in Figure 6. From the graph, it can be observed that the peak of the source current exhibits a growing trend with increasing LET, and with the higher LET, at this point, the circuit feedback is no longer required, leaving only two peaks in the drain node. Additionally, the occurrence time of this secondary peak is before 1.1 × 10^−10^ s, while in Figure 7, the occurrence time of the third peak is between 1.1~1.2 × 10^−10^ s. Therefore, in Figure 4, the second and third peaks correspond to the drift collection process and the SRAM circuit feedback, respectively. The source current also exhibits a period of positive current as the LET value increases. This is because, under high LET values, excessive charges diffuse to the depletion region near the source and are collected by the source. The surplus electrons flow out of the device through the source, resulting in a current entering the device (a positive current value represents current entering the device).

Under high LET values, in addition to the continuously growing main peak, the tail current formed by diffusion also gradually increases with the increase in LET. In theory, the charge collection phenomenon caused by the parasitic bipolar amplification effect will also be continuously enhanced with the increase in LET. However, for the NMOS transistor here, under the LET of 0.5 pC/μm, the drain voltage has already dropped to near 0 V before 1 × 10^−10^ s, and the source voltage is always grounded at 0 V. Therefore, it does not meet the conditions for the activation of the parasitic bipolar amplification effect. The parasitic bipolar amplification effect refers to the disturbance of the substrate or well potential under heavy-ion bombardment conditions, causing the parasitic bipolar transistor formed by the source-drain or substrate-channel to open, leading to an increase in the collected charge at the drain. If it is the reverse parasitic bipolar amplification effect, it will lead to a decrease in the collected charge at the drain. Usually, PMOS corresponds to the parasitic bipolar amplification effect, while NMOS corresponds to the reverse parasitic bipolar amplification effect. Therefore, if the parasitic bipolar amplification effect occurs, the source and drain should either absorb electrons or release electrons. However, when examining the electron current at the source, it is found that both the source and the drain are releasing electrons during this period. This confirms again that the physical meaning of the second peak is the drift collection process.

### 3.2. Charge Collection in PMOS Transistor

**Figure 8:**
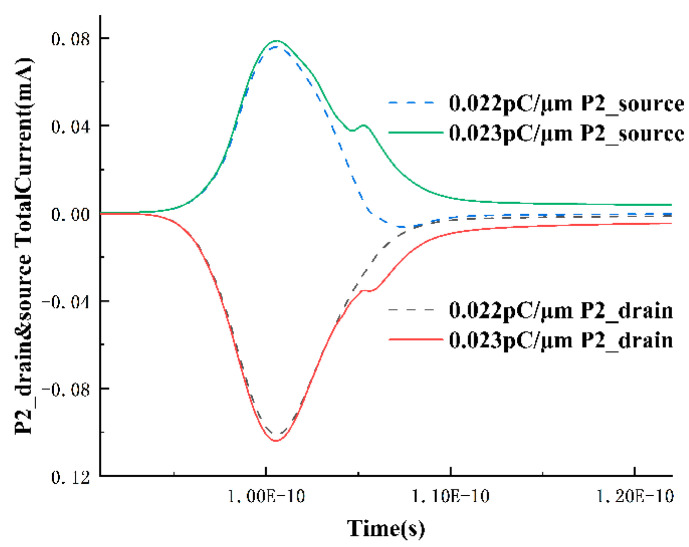
Variation in source and drain currents over time in the P2 transistor.

With the experience gained from analyzing the NMOS transistor, the analysis of the PMOS transistor can be relatively simplified. The source and drain currents of the P2 transistor near the LET threshold are extracted, as shown in Figure 8. At the LET value of 0.022 pC/μm, there is only one peak, but as the LET increases to 0.023 pC/μm and a single-event upset occurs, a secondary peak appears, which, according to the analysis of the NMOS transistor in the previous section, is formed by the circuit feedback. Additionally, it can be observed that when the LET is 0.022 pC/μm, the current value of the source current is negative for a period of time, indicating that some holes have already been collected by the source and flowed out of the device at this point.

**Figure 9:**
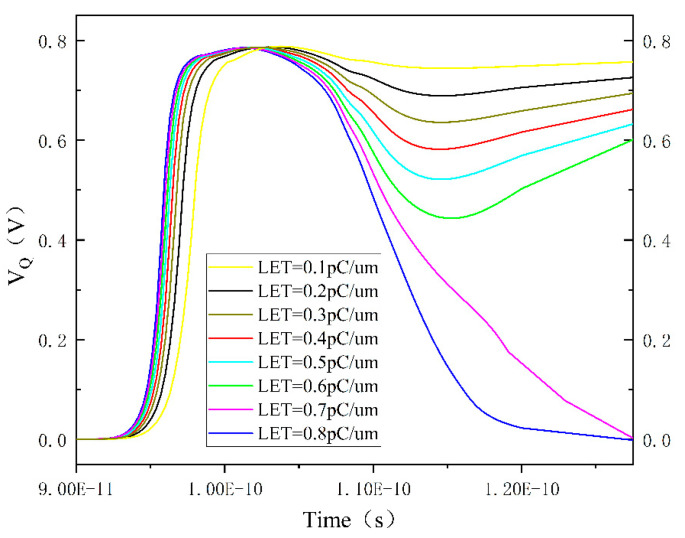
Time-dependent curve of Q-point voltage under high LET conditions.

As the LET value continues to increase, an interesting phenomenon occurs: the drain voltage of the PMOS transistor exhibits a rebound after a single-event upset, as shown in Figure 9. Observing the graph, it can be noted that as the particle LET gradually increases, the trend of the Q-point voltage dropping after reaching a high voltage becomes more pronounced. Until LET = 0.7 pC/um, the Q-point voltage, after a significant drop, does not recover to the high voltage but is lowered again to 0 V within 0.2 ns. In other words, the SRAM cell undergoes two upsets within the 0.2 ns, showing a phenomenon of SRAM cell flip and recovery. The subsequent voltage changes are not shown in the graph, but within the next 30 ns, the curves with LET less than or equal to 0.6 pC/um all return to 0.8 V.

**Figure 10:**
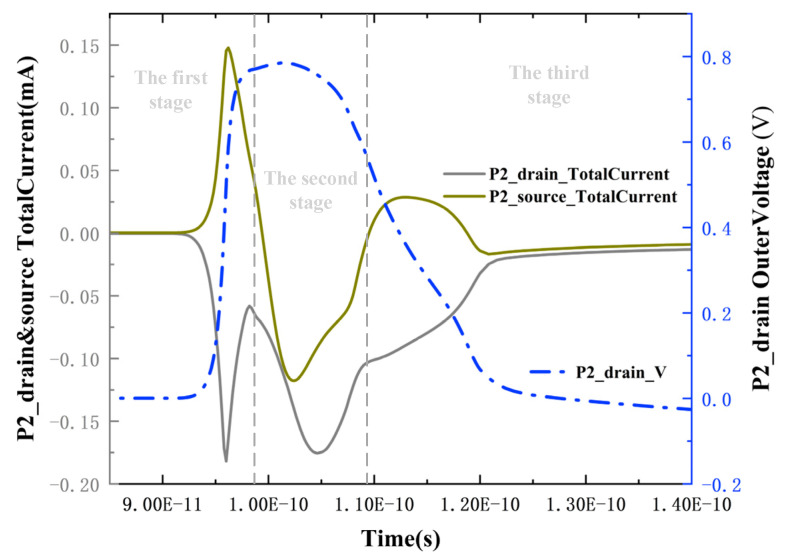
Total current situation of P2 drain and source at LET = 0.7 pC/μm. Positive current values represent current flowing into the device; negative values represent current flowing out of the device.

The first upset process is undoubtedly caused by the PMOS transistor conducting under the influence of heavy ions, leading to the Q-point voltage pulling up. To investigate the second upset process, the total current between the drain and source of P2 at LET = 0.7 pC/μm is extracted, as shown in Figure 10. In the first stage, the drift dominates. During this period, a large number of electron-hole pairs are deposited near the drain, causing the barrier between the drain and source to fail. Source-drain conduction occurs, and the current flows from the source to the drain, corresponding to the phenomenon of rapid voltage increase during this time. Subsequently, in the second stage, the phenomenon of source and drain currents flowing out of the device (negative current values represent current flowing out of the device) occurs. Under the influence of higher LET values, a large number of holes deposited near the drain begin to be collected by the pn junctions near the source and drain through diffusion, forming two currents flowing out of the drain and source, respectively. However, around 108 ps, there is a turning point in the two currents. The source current rapidly decreases and changes from flowing out of the device to flowing into the device, while the decrease in drain current slows down slightly. It can be determined that a new charge collection mechanism is involved at this time.

**Figure 11:**
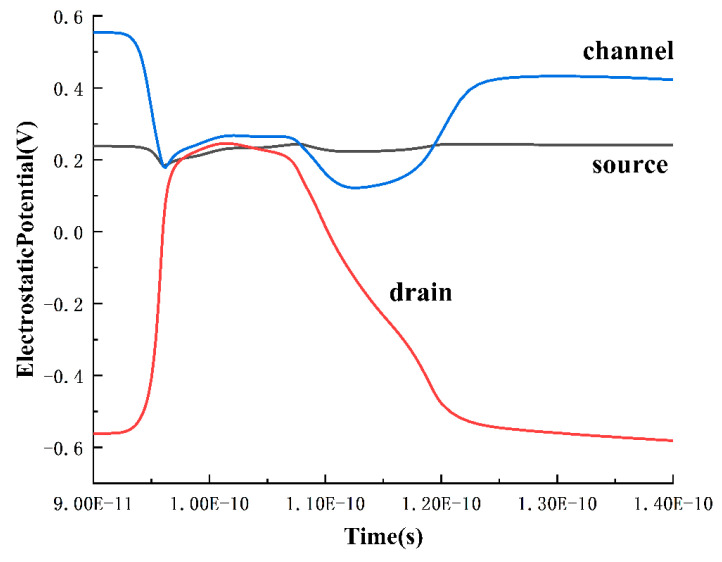
Potential variation of P2 drain, source, and channel at LET = 0.7 pC/μm.

To further analyze the reasons for the changes in current in the third stage, potentials of the source, drain, and channel in the P2 transistor were extracted over time, as shown in Figure 11. During the primary stage of voltage decrease, due to the substantial collection of holes, the potential of the N-channel is lower than that of the source. This results in the forward biasing of the PN junction between the source and the channel, forming a parasitic PNP transistor (source–channel–drain). The PN junction between the drain and the channel remains reverse-biased. As a consequence, the source–drain current transitions from outflow to inflow during this stage, as the forward-biased junction causes the source to emit holes towards the drain. Simultaneously, the drain experiences a reduced rate of current decrease due to the additional injected current. However, from a comprehensive examination of Figure 9 and Figure 10, it is observed that the P2 transistor has not undergone parasitic bipolar amplification at the beginning of the Q-point voltage drop. Additionally, the occurrence of parasitic bipolar amplification in the P2 transistor would only raise the voltage. Therefore, the deceleration observed at 110 ps in the Q-point voltage drop in Figure 10 is not attributable to the P2 transistor.

**Figure 12:**
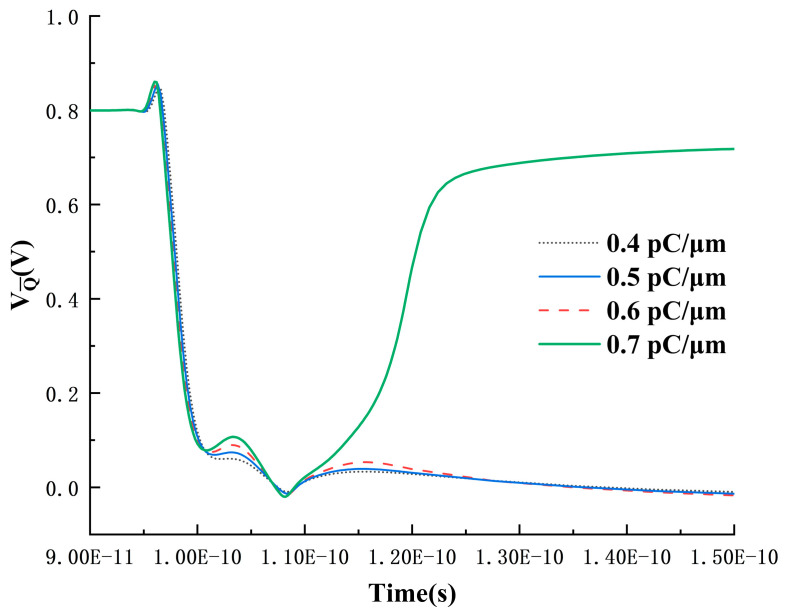
Time-dependent curve of $\bar{Q}$-point voltage under high LET conditions.

Analyzing the circuit structure reveals that, at this point, the only factors causing the Q-point voltage to drop are the conduction of the N2 transistor for pulling down the voltage or the conduction of the P1 transistor for pulling up the voltage. In Figure 9, as the LET value increases, the Q-point voltage uniformly decreases after reaching a high voltage and eventually recovers until, after 0.7 pC/μm, the Q-point voltage reaches the low-voltage noise margin, preventing recovery to the high voltage. The $\bar{Q}$ point voltage variation under high LET values is illustrated in Figure 12, showing a minimal change when the LET value is below 0.7 pC/μm. It rapidly jumps to the high voltage region when LET equals 0.7 pC/μm. Therefore, it can be concluded that the $\bar{Q}$ point voltage rise is caused by the Q-point voltage drop and is conducted through the feedback circuit, ruling out the possibility of the P1 transistor conduction causing the Q-point voltage drop. At this point, it can be confirmed that the Q-point voltage drop is due to the conduction of the N2 transistor pulling it down.

**Figure 13:**
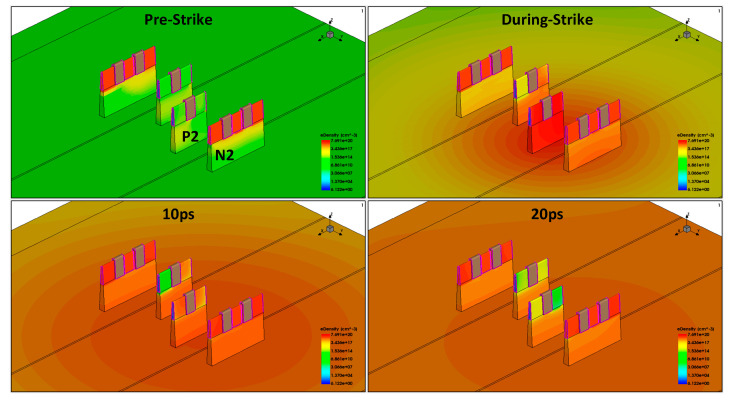
3D TCAD process simulation before ion incident and after 0.01 ns, 10 ps, and 20 ps (the STI is hidden).

The conduction of the N2 transistor requires a substantial number of electrons. The electron concentration distribution in the device before and after heavy-ion impact is depicted in Figure 13. Although initially the electron concentration is mainly distributed near the impact position, after 10 ps post-impact, as the diffusion process progresses, there is a significant electron concentration around each transistor. This is a crucial factor leading to the gradual opening of the N2 transistor. Therefore, the flip-flop and recovery process of the SRAM can be summarized as follows: when the heavy-ion impact position is the P2 transistor, the P2 transistor is initially affected, conducting and pulling up the Q-point voltage. During the voltage pull-up process, a large number of charges deposited through the diffusion process move to the nearby N2 transistor, causing the N2 transistor to conduct again, pulling down the Q-point voltage. It is this diffusion-induced multi-node charge collection process that triggers the upset followed by recovery phenomenon in the SRAM cell.

## 4. Conclusions

Through TCAD simulation, this paper delves into the single-event upset response process in FinFET SRAM cells and analyzes the charge collection principles of NMOS and PMOS transistors at different Linear Energy Transfer (LET) values. The simulation analysis indicates that at low LET values, especially near the LET threshold, the SRAM cell relies on its own feedback circuit to achieve voltage reversal when experiencing a single-event upset. In contrast, at high LET values, the PMOS transistor exhibits not only the traditional drift-diffusion charge collection method but also the occurrence of parasitic bipolar amplification effects. Additionally, multi-node charge collection is identified as a primary factor leading to the phenomenon of the upset followed by recovery during the single-event upset process in SRAM cells. These research findings are crucial for understanding and optimizing the performance of FinFET technology in radiation environments.

## Figures and Tables

**Table 1:** Model Main Parameters.

Main Parameters	Value	Main Parameters	Value
Supply voltage	0.8 V	Substrate Doping Concentration	1.0 × 10^15^ cm^−3^
Fin Height	42 nm	Channel Doping Concentration	1.0 × 10^17^ cm^−3^
Fin Width	10 nm	S and D Doping Concentration (P type)	1.35 × 10^20^ cm^−3^
Lgate	24 nm	S and D Doping Concentration (N type)	7.5 × 10^19^ cm^−3^
STI	60 nm	Gate Oxide Thickness	2 nm

## Data Availability

Data are contained within the article.
